# Potential Oncogenic Effect of the MERTK-Dependent Apoptotic-Cell Clearance Pathway in Starry-Sky B-Cell Lymphoma

**DOI:** 10.3389/fimmu.2020.01759

**Published:** 2020-08-20

**Authors:** Sarah Farnworth-McHugh, Nicole Barth, Lynsey Melville, Margaret Paterson, Catherine Lynch, Pamela Holland, Ian Dransfield, Christopher Gregory

**Affiliations:** ^1^Centre for Inflammation Research, University of Edinburgh, Edinburgh, United Kingdom; ^2^School of Chemistry, University of Edinburgh, Edinburgh, United Kingdom

**Keywords:** non-Hodgkin lymphoma, apoptosis, TYRO3-AXL-MERTK, receptor tyrosine kinase, macrophage, phagocytosis

## Abstract

The histological architecture of certain aggressive B-cell lymphomas (prototypically Burkitt's lymphoma, BL) is characterized by a “starry-sky” (SS) appearance. This is caused by tumor-associated macrophages (TAMs), which appear in standard histological preparations as “stars” in a darkly stained “sky” of lymphoma cells. SS-TAMs accumulate in response to constitutive apoptosis in these tumors and are activated by the apoptotic tumor cells to a pro-oncogenic phenotype. The extent to which SS-TAMs contribute to lymphoma growth through responses generated by interactions with apoptotic tumor cells is unknown. Here, we demonstrate a role for the receptor tyrosine kinase, MERTK, in the oncogenic activity of SS-TAMs. We show that MERTK expression is largely restricted to the macrophages of human BL and of murine models of SS B-cell lymphoma and that it is upregulated in SS-TAMs as compared to the germinal center or paracortical macrophages of normal lymph nodes. Our results further demonstrate that MERTK is active in the phagocytosis of apoptotic lymphoma cells by macrophages and, most significantly, that SS lymphoma growth is markedly inhibited in *Mertk*^−/−^ mice. These results point toward the MERTK apoptotic-cell clearance/response pathway playing a key role in growth of aggressive B-cell lymphoma and identifies MERTK as a novel potential antilymphoma target.

## Introduction

Constitutive tumor-cell apoptosis is high in aggressive cancers, including non-Hodgkin lymphoma (1). Emerging evidence indicates that responses to apoptosis in the tumor microenvironment can promote cancer growth, not only in primary tumors, but also in post-therapeutic relapse (2–7). In SS lymphomas, apoptosis is highly prominent in standard biopsy preparations, and a key response to apoptotic tumor cells is the accumulation of TAMs, together with their activation to a pro-oncogenic phenotype (5). It remains unclear however, how apoptotic tumor cells activate TAMs to help promote net tumor growth. Here, we focus on MERTK, a member of the TYRO3/AXL/MERTK family of receptor tyrosine kinases, which regulate tissue development and homeostasis via two mechanistically related immunosuppressive functions: the clearance of cells undergoing apoptosis and anti-inflammatory signaling (8–10). It is becoming increasingly evident that the TYRO3/AXL/MERTK signaling axis has oncogenic properties in a wide range of cancers. In hematopoietic malignancies, although individual components of this axis are expressed by tumor cells and/or TAMs (11–14), their role(s) in the pathogenesis of these cancers have not been defined. MERTK is a well-established phagocyte receptor for clearance of apoptotic cells (8–10) operating via its ligands, GAS6, and Protein S (PROS1), which bridge the receptor to phosphatidylserine (PS) exposed at apoptotic cell surfaces. Inhibition of MERTK leads to persistence of apoptotic cells, especially in the germinal centers of lymphoid follicles and to the associated emergence of autoimmune disease symptoms (10, 15, 16). Preferential expression of *MERTK* and *GAS6* are associated with reparatory, M2-like macrophage polarization (5, 17), which is typical of wound-healing macrophages and of TAMs (18). In the present brief investigation, we tested the hypothesis that MERTK is involved in the clearance of apoptotic lymphoma cells by SS-TAMs and that it is important for the growth of aggressive, SS lymphoma.

## Materials and Methods

### Cell Lines and Animal Models

The BL cell line, BL2 was derived from a sporadic, Epstein-Barr virus–negative case of BL (19). The THP-1 line was established from a patient with monocytic leukemia (20). Both lines were cultured *in vitro* as we have previously described (21). BL2 cells were xenografted subcutaneously to SCID mice according to our established methods (5) and formed aggressive, starry-sky tumors. We previously derived the MycEd1 cell line (5) from an aggressive starry-sky B-cell lymphoma of a male C57BL/6 mouse carrying the λ*-MYC* transgene (22). MycEd1 cells were cultured *in vitro* and also used in transplantation experiments using our previously established protocols (5) in wild-type (WT) and *Mertk*^−/−^ C57BL/6 mice (23) (kindly provided by Dr. Greg Lemke, The Salk Institute for Biological Studies). MycEd1 tumor growth was assessed following subcutaneous injection of 5 × 10^6^ viable Myc-Ed1 cells into 6- to 12-week-old male WT or *Mertk*^−/−^ mice. Mice were observed daily, and growth of tumors was monitored using calipers. In all experiments, mice were humanely sacrificed either (a) when tumors reached maximal dimensions according to the UK Animals (Scientific Procedures) Act 1986 regulations or (b) day 20 post-injection, whichever was the sooner.

### Gene Expression Analysis

“*In situ* transcriptomics” of SS-TAMs from BL (BL2) xenografts, lymph node germinal center (GC), and paracortical macrophages was performed following laser capture microdissection of macrophages exactly as described (5). λ-MYC [MycEd1 cell line, see (5, 24)] gene expression was carried out on Affymetrix Mouse Gene 2.1 GeneChip arrays. Gene expression analysis of BL2 and primary human dendritic cells [DC, myeloid positive control cells, prepared as we have described previously (25)] was carried out on Affymetrix Human Genome U133 Plus 2.0 arrays. Data were processed in R and normalized with RMA. Real time RT-PCR was carried out as follows: RNA was isolated using RNeasy Mini Kit (Qiagen), DNAse-treated and reverse-transcribed into cDNA using SuperScript III First-Strand Synthesis SuperMix for qRT-PCR (Life Technologies). Real-time PCR was performed using Fast SYBR Green Master Mix (Applied Biosystems). qPCR was performed using an ABI 7900 Real Time PCR Machine with ABI SDS (Sequence Detection System) software.

### Immunocytochemistry and Immunohistochemistry

Human cells (viable or apoptotic BL2 or THP1) were labeled with antihuman MERTK-PE (clone 125518) R&D FAB8912P (isotype mouse IgG2b, κ) or mouse PE-conjugated isotype control (clone 11711) R&D IC002P and mouse cells (MycEd1 or bone marrow–derived macrophages, BMDMs) with antimouse MERTK-PE (clone 108928) R&D FAB5912P (isotype Rat IgG2A) or Rat IgG2A PE-conjugated isotype control (clone 54447) R&D IC006P prior to flow cytometric analysis.

Formalin-fixed, paraffin-embedded tissues were sectioned and stained with standard hematoxylin and eosin or were used in immunohistochemistry (IHC) as described (5). Sections were labeled with monoclonal mouse antihuman CD68 clone PG-M1 (Dako M0876) or polyclonal goat antihuman MERTK (R&D AF891), and subsequently with either goat antimouse IgG, biotinylated (Vector #BA-9200) or horse antigoat IgG, biotinylated (Vector #BA-9500). Following incubation with Vectastain Elite ABC Reagent and DAB (Vector SK-4100), samples were counterstained in hematoxylin.

### Immunoblotting

WT murine BMDMs treated with or without 200 nM dexamethasone for 24 h, MycEd1 cells, undifferentiated THP1, THP1 cells differentiated with PMA (100 ng/ml) for 2 days, and BL2 cells were lysed in reducing cell lysis buffer, 5 μg lysate were loaded per well on a 4–12% Bis-Tris NuPAGE gel, run under reducing conditions then transferred to Hybond-P. Membrane was blocked in 5% BSA then incubated in 1:1,000 goat antimouse MERTK (R&D #AF591) or 1:1,000 goat antihuman MERTK (R&D #AF891), for mouse and human lysates, respectively, in 5% BSA overnight at 4°C. Membranes were then incubated in 1:5,000 donkey antigoat IgG-peroxidase (Jackson ImmunoResearch #705-035-003) in 5% milk for 1 h at room temperature and then developed using ECL.

### Apoptosis, Ligand Binding, and Phagocytic Clearance of Apoptotic Cells

Human monocyte-derived macrophages (HMDMs) and murine BMDMs were prepared as described (24, 26). In brief, BMDMs were prepared from the femurs of 8- to 12-week-old mice and cultured for 7–8 days with 100 ng/ml rhM-CSF (R&D Systems, Abingdon, UK) on bacteriological-grade Petri dishes. HMDMs were prepared from peripheral blood monocytes enriched using the pan monocyte isolation kit, human (MACS Miltenyi #130-096-537) and cultured in 2% human AB serum for 7 days. Apoptosis was induced in lymphoma cells by UV-treatment and assessed by flow cytometry following annexin V (AxV)/Sytox Blue staining (5).

Ligand binding: GAS6 and PROS1 proteins were coupled using the Cy5 Antibody Labeling Kit (GE Healthcare, Buckinghamshire, UK). Briefly, protein to be labeled (at 1.0–1.2 mg/ml) was exchanged into 100 mM sodium hydrogen carbonate buffer (pH 8.3) and incubated with Cy5 mono-reactive dye pack for 30 min in the dark at room temperature as recommended by the manufacturer (www.GELifesciences.com). The reaction was terminated by addition of glycine to a final concentration of 50 mM, and then protein was buffer-exchanged into PBS. The degree of protein labeling was estimated from measurement of absorbance at 280 and 650 nm (Cy5) and was routinely found to be between 2.5 and 3.5 moles of dye/mole of protein. Functionality of labeled protein was assessed by testing the potential to induce MERTK phosphorylation and the capacity to confer MERTK-dependent phagocytosis of apoptotic cells (data not shown). Ligand binding to lymphoma cells (±UV treatment) was carried out along with Annexin-V-488 and Sytox Blue labeling (Life Technologies) in 20 mM HEPES buffer containing 140 mM NaCl, 0.1% BSA and 2 mM CaCl_2_ followed by flow cytometry. To control for nonspecific binding of the fluorescent proteins, binding was carried out using buffer in which the CaCl_2_ was substituted by 2.5 mM EDTA (specific binding of Annexin V, GAS6, and PROS1 all require Ca^2+^). Phagocytosis of apoptotic cells was assayed objectively by flow cytometry using well-characterized, established methods (27). In these assays, apoptotic cells were stained with 1 μM pHrodo (Life Technologies #P36600) and macrophages were labeled with 0.5 μg/mL CellTrace Far Red (Life Technologies #C34564). Phagocytic macrophages exhibit enhanced pHrodo fluorescence following internalization of labeled apoptotic lymphoma cells into the acidic environment of phagosomes. GAS6 and gla-less GAS6 were kindly provided by Dr. Erin Lew, The Salk Institute, and the gla-less PROS1 was kindly provided by Mary Jo Heeb (Scripps Research Institute) (28); PROS1 (HPS 4590AL) was purchased from Enzyme Research Laboratories (South Bend, IN, USA).

## Results

### MERTK Is Expressed Preferentially by Macrophages in SS-Lymphoma

**Figure 1:**
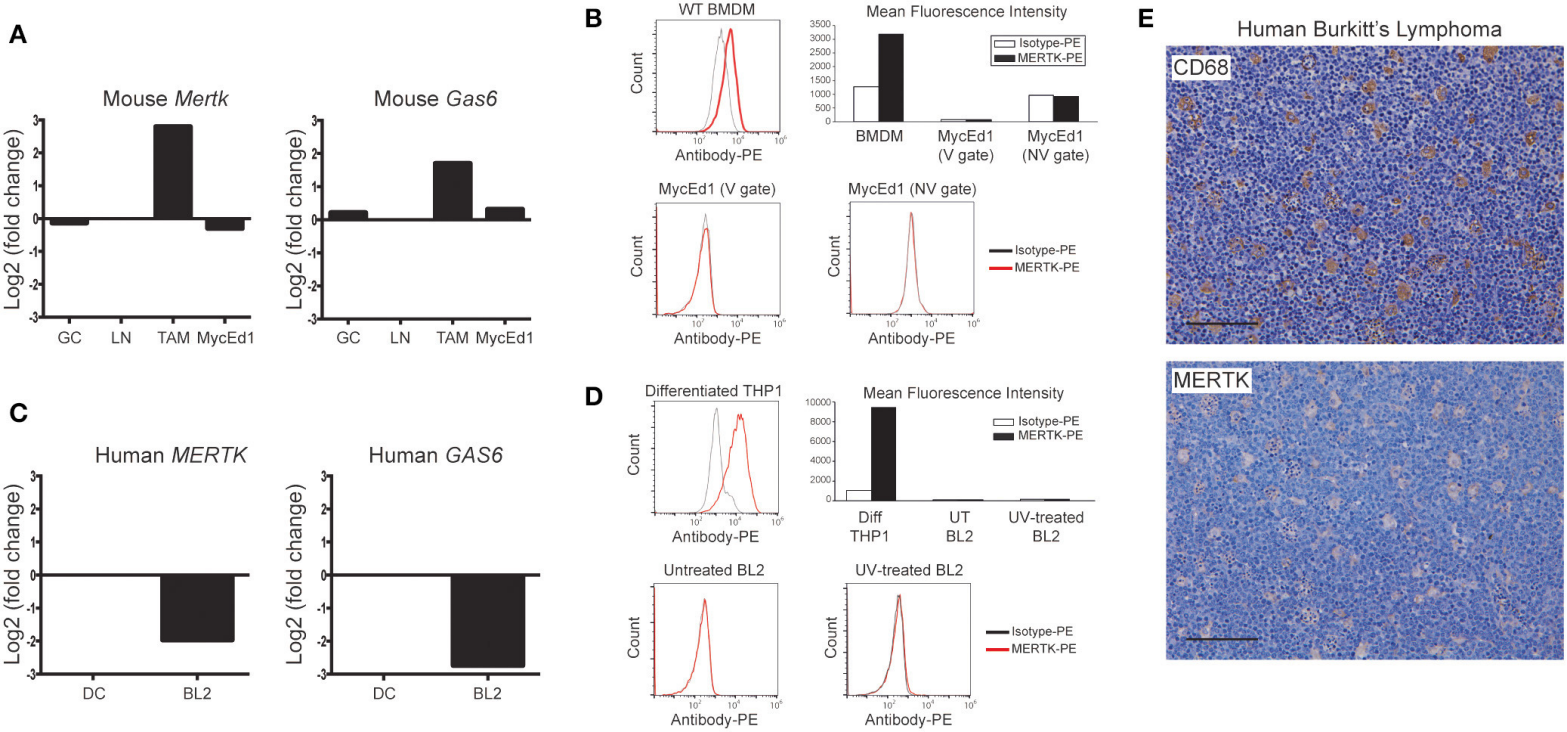
Preferential expression of MERTK by macrophages in SS-NHL. **(A)** *Mertk* and *Gas6* expression are low/absent in murine SS-NHL cells (MycEd1) relative to SS-TAMs. Mouse gene expression data for *Mertk* and *Gas6* are shown as log_2_ fold-change relative to paracortical lymph node macrophages (LN). Expression is also shown for tingible body macrophages of lymph node germinal centers (GC). **(B)** Absence of mouse MERTK protein expression from murine MycEd1 lymphoma cells. Untreated MycEd1 cells were analyzed by flow cytometry (MycEd1 cultures have a high level of constitutive apoptosis). WT BMDMs were used as positive control cells. **(C)** Human *MER*TK and *GAS6* gene expression data in BL2 cells shown relative to dendritic cells (DC). **(D)** Human MERTK protein as analyzed by flow cytometry is absent from BL2 tumor cells; differentiated THP-1 cells were used as positive controls. BL2 cells were either left untreated or were UV-treated (300 mJ/cm^2^) and incubated for 3 h at 37°C to become apoptotic (cells were 60% apoptotic by AxV/Sytox blue). Representative flow cytometric analyses are shown; gating strategies, including those delineating viable (V) and nonviable (NV) cells, are shown in Supplementary Figure 1D. **(E)** SS-TAMs in BL are CD68 and MERTK positive. Representative micrographs, scale bar = 100 μm.

Throughout these investigations, we used well-characterized models of murine and human SS-lymphoma (MycEd1 and BL2, respectively). Through *in situ* transcriptomics of SS-TAMs of BL xenografts (5), we initially noted increased expression by TAMs of several members of the TYRO3/AXL/MERTK axis, including *Mertk* and *Gas6*, as compared with tingible body macrophages from germinal centers or paracortical lymph node macrophages. By contrast, expression of mouse *Mertk* and *Gas6* by murine lymphoma cells (MycEd1) was low or absent (Figure 1A). Absence of MERTK protein expression by murine lymphoma cells was confirmed by flow cytometry (Figure 1B), by immunoblotting (Supplementary Figure 1A) and by real-time RT-PCR (Supplementary Figure 1B). Focusing on human BL, we found that *MERTK* and *GAS6* gene expression was low or absent from BL2 cells, relative to DCs (Figure 1C) and that MERTK protein expression was absent from BL2 cells *in vitro* (Figure 1D) and largely restricted to SS-TAMs *in vivo* (Figure 1E), confirming the transcriptomics data. Furthermore, TAMs engulfing apoptotic BL cells *in situ* tended to be MERTK^+^ (Figure 1E). Absence of MERTK expression by BL2 cells was also confirmed by immunoblotting (Supplementary Figure 1C).

### MERTK and Its Ligands Are Required for Efficient Clearance of Apoptotic Human Lymphoma Cells by Macrophages

**Figure 2:**
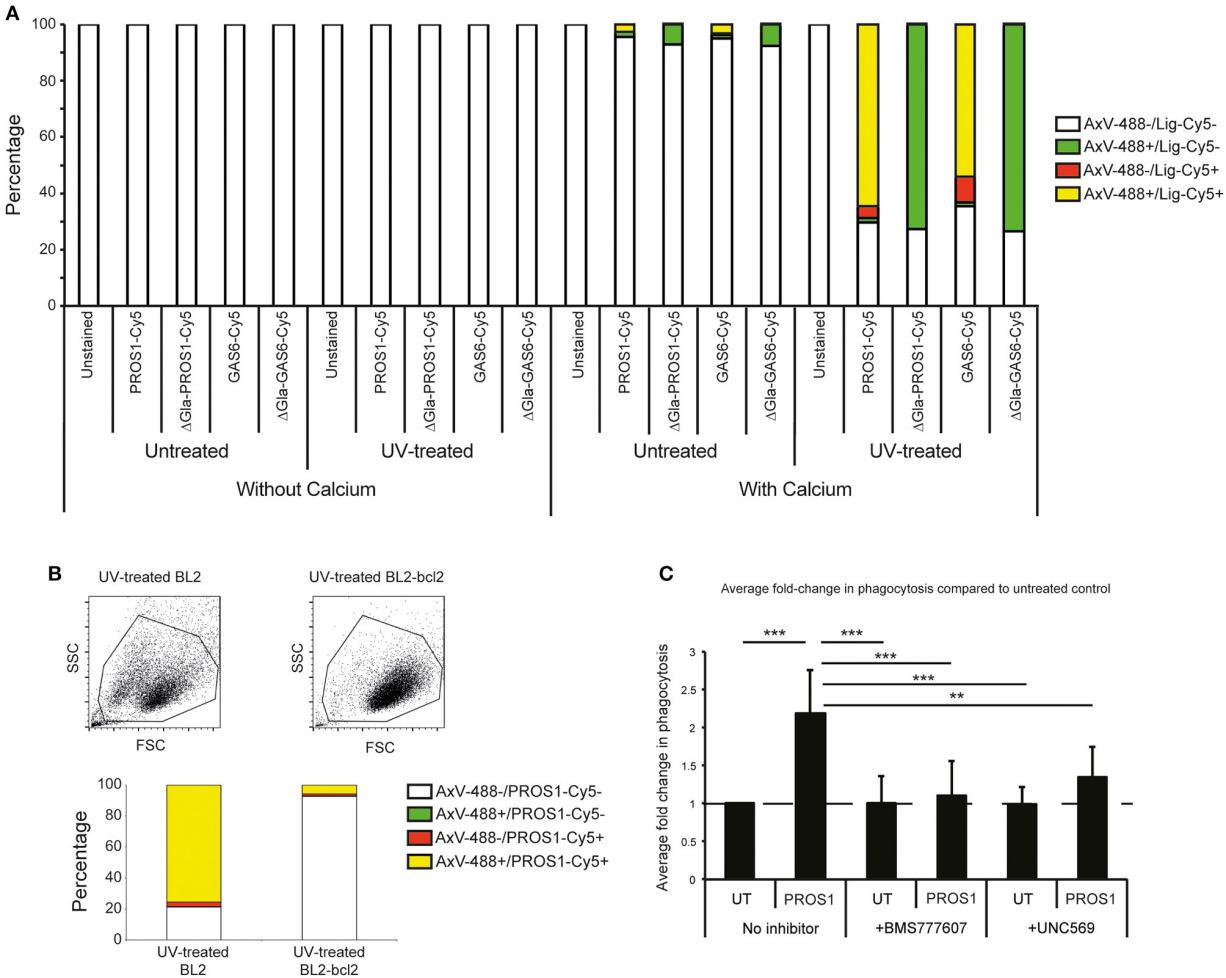
Requirement for MERTK and its ligands in macrophage clearance of apoptotic human lymphoma cells. **(A)** MERTK ligands PROS1 and GAS6 preferentially bind apoptotic BL2 cells in a Ca^2+^ and gla-domain-dependent manner. Flow cytometric analyses of untreated (low levels of constitutive apoptosis) or UV-treated (high levels of apoptosis) BL2 cells incubated with Cy5-conjugated ligands (PROS1-Cy5, PROS1-Gla-less-Cy5, GAS6-Cy5, and GAS6-Gla-less-Cy5) in the presence of 2 mM CaCl_2_ or 2.5 mM EDTA (“without calcium”) and with AxV-488 for dual staining. The without-calcium samples were included to control for nonspecific binding of the fluorescent proteins (see section Materials and Methods). **(B)** MERTK ligand, PROS1 preferentially binds apoptotic BL2 cells as compared with apoptosis-resistant BL2-bcl2 cells. BL2 cells were UV-treated and incubated for 5 h at 37°C to become apoptotic. BL2-bcl2 cells undergoing the same procedure were used as a viable control. Cells were washed and resuspended in buffer with 2 mM CaCl_2_. Cells were labeled with AxV-488, PROS1-Cy5 or both. Note that nonirradiated control BL2-bcl-2 cells bound neither AxV, nor MERTK ligands (not shown). **(C)** Phagocytosis of apoptotic BL2 cells by HMDMs is enhanced by PROS1 and inhibited with MERTK inhibitors (BMS 777607 and UNC569). Apoptotic BL2 cells were stained with pHrodo and HMDMs were labeled with CellTrace Far Red. Stained HMDMs were pretreated with either 500 nM BMS 77760 or 2.5 μM UNC569 for 40 min prior to phagocytosis assay. Apoptotic cells were coincubated with HMDMs at a ratio of 7:1 ± 25 nM PROS1. Inhibitors were diluted during the coculture to 100 nM and 0.5 μM for BMS 77760 and UNC569, respectively. BL2 and HMDMs were cocultured for 40 min prior to lifting the HMDMs using trypsin/EDTA and analysis by flow cytometry as described (27). Data shown are mean fold changes of phagocytic macrophages ± SEM. **p < 0.01, ***p < 0.001 One-way ANOVA with Bonferroni post-test (*n* = 6–8).

To determine the potential role of MERTK and its ligands in phagocytic clearance of apoptotic BL2 cells, we next tested whether the ligands GAS6 and PROS1 are capable of opsonizing these cells. As shown in Figures 2A,B, both murine recombinant Gas6 (which is known to be a good ligand for human MERTK) and PROS1, purified from human plasma, bound strongly to apoptotic BL2 cells in a manner dependent on the PS-binding Gla domains of the ligands. Furthermore, we used PROS1 to demonstrate increased phagocytosis of apoptotic BL2 cells by HMDMs *in vitro* (Figure 2C). The PROS1-enhanced phagocytosis was suppressed by the MERTK kinase inhibitor UNC569 and by the c-MET inhibitor BMS777607, which, at the concentration used, also has MERTK inhibitory activity (Figure 2C). These results confirm that MERTK signaling supports phagocytic clearance of apoptotic BL2 cells by macrophages and, together with the *in situ* expression analyses, are consistent with the notion that engulfment of these cells by SS-TAMs is MERTK-dependent.

### Murine Macrophage MERTK Is Required for Clearance of Apoptotic Lymphoma Cells and for SS-Lymphoma Growth *in vivo*

**Figure 3:**
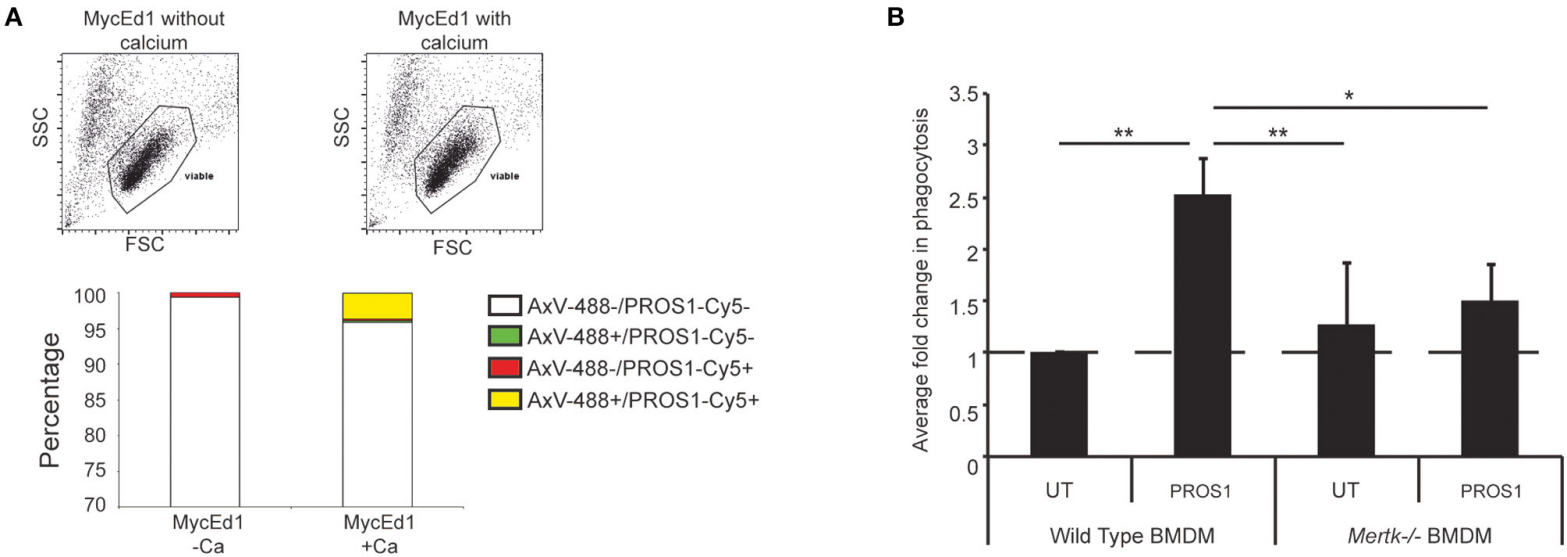
Requirement for MERTK in macrophage clearance of apoptotic murine lymphoma cells. **(A)** Flow cytometric analyses demonstrating that MERTK ligand, PROS1 binds apoptotic murine lymphoma cells (MycEd1) in a Ca^2+^-dependent manner. MycEd1 cells ± UV treatment were incubated with PROS1-Cy5 in the presence of 2 mM CaCl_2_ (+Ca^2+^) or 2.5 mM EDTA (–Ca^2+^) and with AxV-488 for dual staining. The “viable” scatter gate is a well-established gate used to reliably detect apoptotic lymphoma cells prior to plasma membrane permeabilization (29). Nonirradiated control MycEd1 cells bound neither AxV nor PROS1 (not shown). **(B)** PROS1 enhances phagocytosis of apoptotic MycEd1 cells by wild-type but not *Mertk*^−/−^ BMDMs. BMDMs from wild-type and *Mertk*^−/−^ mice were coincubated with pHrodo-labeled UV-treated MycEd1 cells in the presence or absence of 25nM PROS1 for 45 min at 37°C. Data shown are mean fold changes of phagocytic macrophages ± SEM. *p < 0.05, **p < 0.01 One-way ANOVA with Bonferroni post-test (*n* = 3–4).

**Figure 4:**
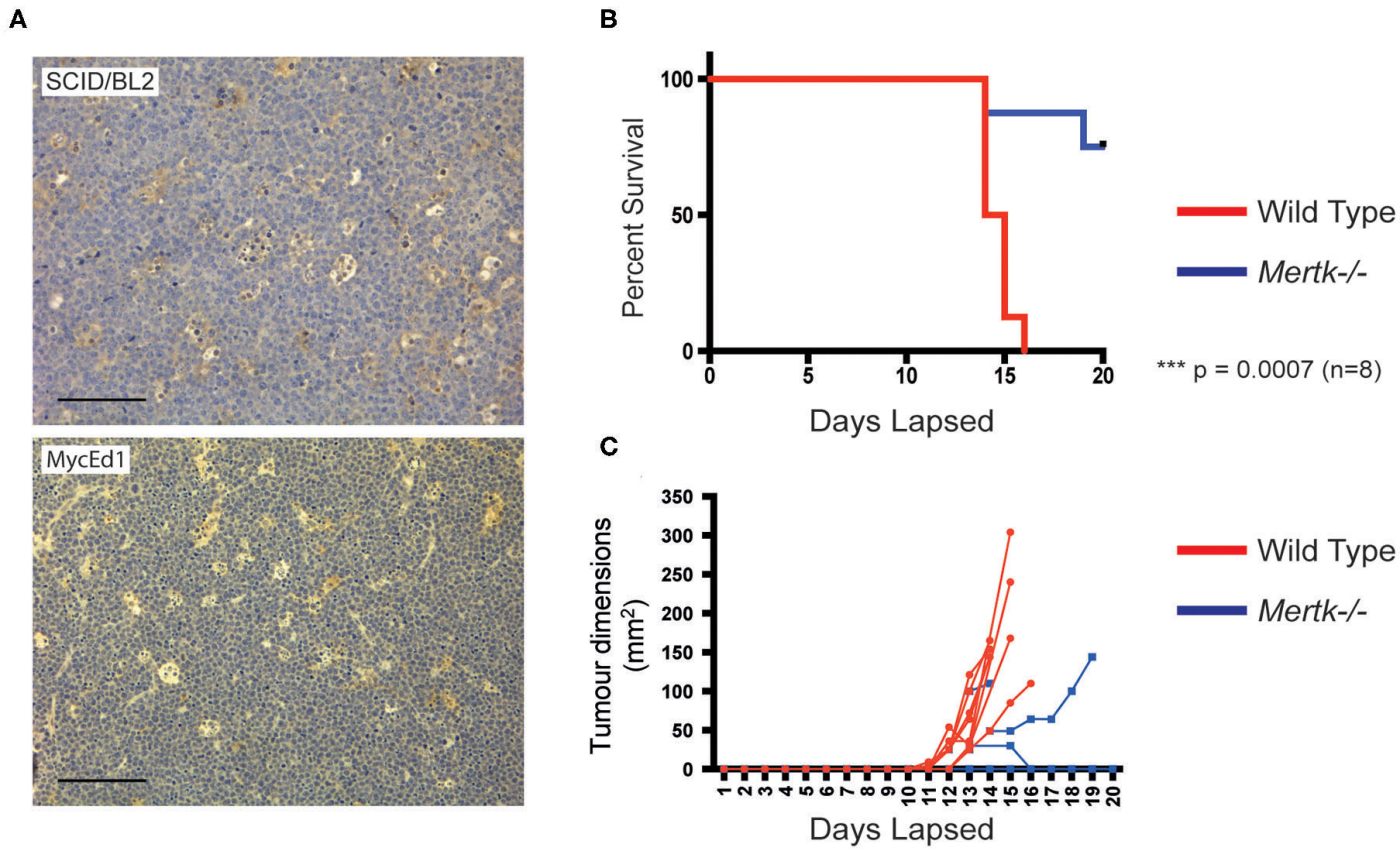
Growth of aggressive SS lymphoma requires Mertk. **(A)** SS-TAMs in mouse model lymphomas are Mertk-positive. Immunohistochemistry of mouse MycEd1 tumors or BL2 xenograft tumors labeled with antimouse Mertk. Scale bar = 100 μm. Representative images. Survival is enhanced **(B)** in parallel with inhibition of MycEd1 tumor growth **(C)** in *Mertk*^−/−^ mice. Male *Mertk*^−/−^ and aged-matched WT littermate control C57BL/6 mice were injected subcutaneously with 5 × 10^5^ MycEd1 lymphoma cells. Mice were observed daily and growth of tumors was monitored using calipers. *P* < 0.001 Mantel-Cox log rank test.

Because MERTK activation by apoptotic lymphoma cells may provide anti-inflammatory and immunosuppressive signals that promote tumor growth, we next tested the requirement for MERTK in an aggressive, preclinical transgenic murine SS lymphoma model, λ*-MYC* (22), which we have used previously using our derived MycEd1 line (5). We found that, just as in human BL, MycEd1 cells became MERTK ligand binding when they underwent apoptosis (Figure 3A), and phagocytosis by macrophages was demonstrably MERTK-dependent (Figure 3B). Similar to BL2 xenografts in mice (Figure 4A, upper panel), immunohistochemical expression of murine MERTK in MycEd1 tumors was mainly by stromal cells, notably SS-TAMs, rather than by the tumor cells themselves (Figure 4A, lower panel). This reflected expression profiling and flow cytometric analyses of MycEd1 cells, which indicated little or no expression of *Mertk* RNA or MERTK protein, respectively (Figures 1A,B and Supplementary Figures 1A,B). Strikingly, growth of MycEd1 tumors *in vivo* was found to be very strongly dependent on *Mertk* (Figures 4B,C).

## Discussion

These results demonstrate a positive, causative link between MERTK expression and growth capacity of SS NHL, at least in the exemplar models studied here. In the context of the proven ability of apoptotic lymphoma cells ultimately to facilitate SS tumor growth, we propose that inhibition of MERTK may be helpful in combination with apoptosis-inducing antilymphoma therapeutics. The present study does not elucidate the detailed mechanism(s) by which MERTK controls SS lymphoma growth. Based on the evidence presented, taken together with published activity of MERTK in immunosuppressive signaling following engagement of appropriately opsonized apoptotic cells (via GAS6 and PROS1), we suggest that interactions between apoptotic lymphoma cells and MERTK-expressing stromal/immune cells of the tumor lie at the root of the mechanism. These may stimulate suppression of antitumor immunity or alternative, trophic responses, such as growth factor production or angiogenesis. In these contexts, it is noteworthy that *Gas6* is upregulated in SS-TAMs, at least in BL2 xenografts (Figure 1A), suggesting that these macrophages of the lymphoma microenvironment are armed with both receptor and ligand for such responses.

Intriguingly, amelioration of λ*-MYC* SS lymphoma growth in the *Mertk*^−/−^ mice phenocopies mice deficient in *Gals3* [galectin-3, see (24)], suggesting that MERTK and galectin-3 could provide different components in a common, pro-oncogenic mechanism. This possibility is supported by evidence that galectin-3 has been implicated both in apoptotic cell clearance by macrophages (30, 31) and as a ligand for MERTK (32). However, we have been unable to demonstrate the latter capacity in relation to apoptotic lymphoma cells (our unpublished observations). Given the capability of galectin-3 to support M2-like activation of macrophages, including TAMs (30, 33), our results are consistent with MERTK and galectin-3 each being required for critical, possibly independent pathways in pro-oncogenic TAM activation. In order to understand in further detail the possible roles of MERTK in the stromal/immune microenvironment of SS-lymphoma, several aspects of the work reported here merit further investigation. These include extended studies of the function of MERTK in polarizing TAM activation (especially including genes like *MRC1, MSR1*, and *LRP1*) that we have previously found to be upregulated in SS-TAMs engaged in engulfment of apoptotic cells as well as investigations into the importance of MERTK expression and activity in other immune/stromal cells. Furthermore, other TYRO3/AXL/MERTK family members, notably AXL, may be important in this regard.

In conclusion, the work presented here provides a strong rationale for the TYRO3/AXL/MERTK axis, notably MERTK, to be targeted in antilymphoma therapy. Further studies in additional lymphoma models will be required to prove the generality of these results and to elucidate in detail the underlying mechanisms that support MERTK-dependent NHL growth.

## Data Availability Statement

The datasets generated for this study can be found in the GEO database, the accession number for datasets analyzed in this study is GSE64366.

## Ethics Statement

The animal studies were approved by the University of Edinburgh Animal Welfare and Ethical Review Body and under the UK Animals (Scientific Procedures) Act 1986 Project Licence number PPL 70/8139.

## Author Contributions

SF-M, NB, LM, MP, CL, and PH performed research and analyzed data. CG and ID designed the research and analyzed data. CG, ID, and SF-M wrote the manuscript with input from other authors. All authors contributed to the article and approved the submitted version.

## Conflict of Interest

The authors declare that the research was conducted in the absence of any commercial or financial relationships that could be construed as a potential conflict of interest.

## Supplementary Material

Supplementary Figure 1 Untreated MycEd1 cells were analyzed by **(A)** Western blotting for MERTK protein or **(B)** real-time PCR for *Mertk* message. WT BMDMs with or without 24 h 200 nM dexamethasone treatment were used as positive control cells for Western blotting. WT and *Mertk*^−/−^ BMDMs were used as positive and negative controls, respectively, for real-time PCR. **(C)** BL2 cells were analyzed for MERTK by Western blotting. Differentiated THP1 cells were used as positive controls. **(D,E)** Scatter plots showing the gating used in Figures 1B,D, respectively.
