# Supplementary figures and images for: Potential Oncogenic Effect of the MERTK-Dependent Apoptotic-Cell Clearance Pathway in Starry-Sky B-Cell Lymphoma
Source: Front Immunol. 2020 Aug 20;11:1759. doi: 10.3389/fimmu.2020.01759 (PMC7468413; doi:10.3389/fimmu.2020.01759)

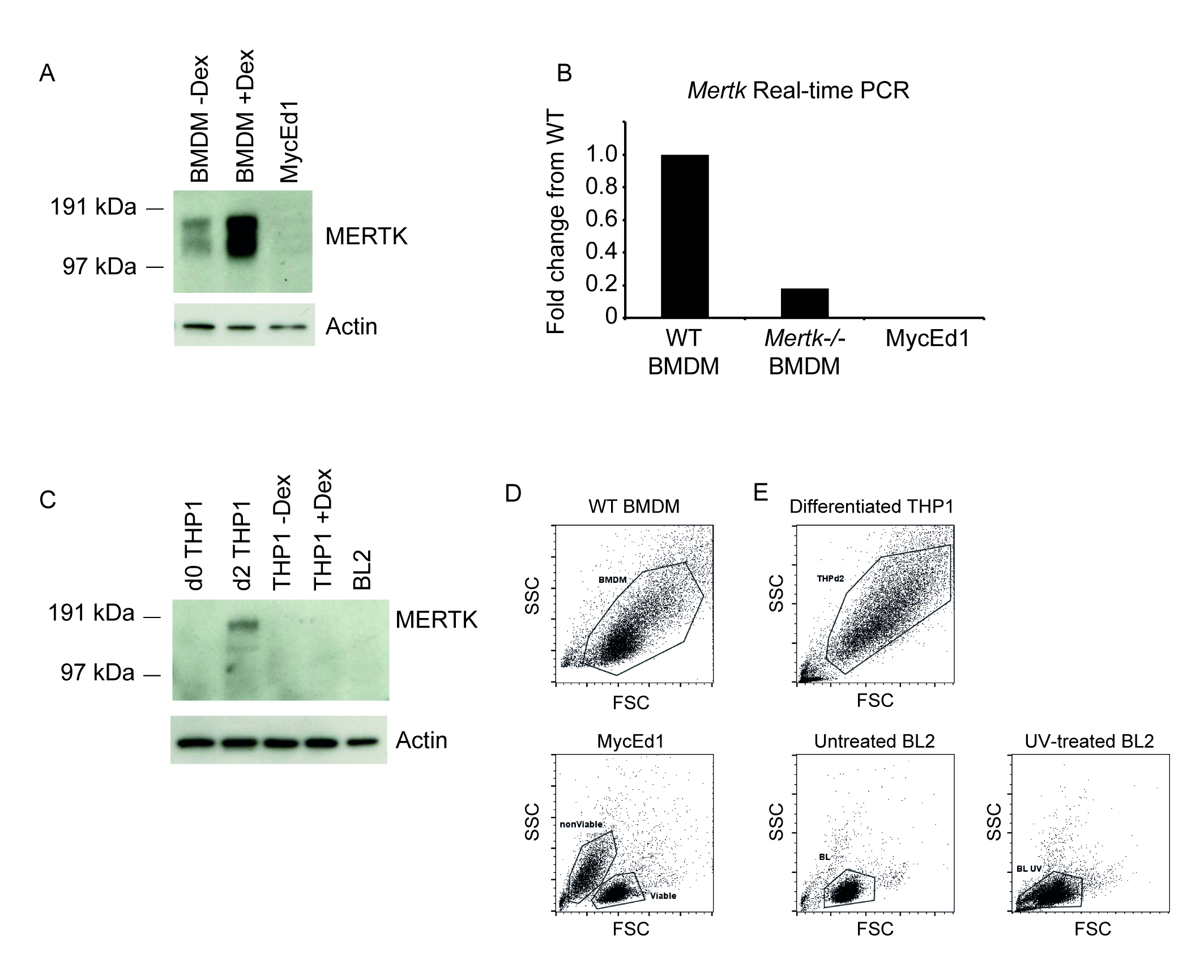

Supplement: Supplementary Figure 1 — Untreated MycEd1 cells were analyzed by (A) Western blotting for MERTK protein or (B) real-time PCR for Mertk message. WT BMDMs with or without 24 h 200 nM dexamethasone treatment were used as positive control cells for Western blotting. WT and Mertk−/− BMDMs were used as positive and negative controls, respectively, for real-time PCR. (C) BL2 cells were analyzed for MERTK by Western blotting. Differentiated THP1 cells were used as positive controls. (D,E) Scatter plots showing the gating used in Figures 1B,D, respectively. [file Image_1.tif]
